# A Novel Method for Measuring the Diffusion, Partition and Convective Mass Transfer Coefficients of Formaldehyde and VOC in Building Materials

**DOI:** 10.1371/journal.pone.0049342

**Published:** 2012-11-07

**Authors:** Jianyin Xiong, Shaodan Huang, Yinping Zhang

**Affiliations:** 1 School of Mechanical Engineering, Beijing Institute of Technology, Beijing, China; 2 Department of Building Science, Tsinghua University, Beijing, China; King’s College London, United Kingdom

## Abstract

The diffusion coefficient (*D*_m_) and material/air partition coefficient (*K*) are two key parameters characterizing the formaldehyde and volatile organic compounds (VOC) sorption behavior in building materials. By virtue of the sorption process in airtight chamber, this paper proposes a novel method to measure the two key parameters, as well as the convective mass transfer coefficient (*h*_m_). Compared to traditional methods, it has the following merits: (1) the *K*, *D*_m_ and *h*_m_ can be simultaneously obtained, thus is convenient to use; (2) it is time-saving, just one sorption process in airtight chamber is required; (3) the determination of *h*_m_ is based on the formaldehyde and VOC concentration data in the test chamber rather than the generally used empirical correlations obtained from the heat and mass transfer analogy, thus is more accurate and can be regarded as a significant improvement. The present method is applied to measure the three parameters by treating the experimental data in the literature, and good results are obtained, which validates the effectiveness of the method. Our new method also provides a potential pathway for measuring *h*_m_ of semi-volatile organic compounds (SVOC) by using that of VOC.

## Introduction

Indoor air pollution from formaldehyde and volatile organic compounds (VOC) is known to pose an adverse threaten to human health [1]–[3]. Formaldehyde, which is regarded as a human carcinogen [4], is of particular concern [5]. Building materials, which have been acknowledged as major formaldehyde and VOC sources in indoor environment, there is also evidence that it can affect the transport and removal of the pollutants by sorption and desorption, i.e., reducing the peak concentration while prolonging the presence. The re-emission of sorbed formaldehyde and VOC can dramatically elevate indoor concentrations for months or even years [6]–[8]. Therefore, knowledge of formaldehyde and VOC sorption in building materials is of great importance.

For the characterization of formaldehyde and VOC sorption in building materials, two general physical models are used, i.e., the first order adsorption/desorption rate model and the equilibrium-interface model [9]–[17]. Although the first order adsorption/desorption rate model is simple and easy to use, the adsorption and desorption constants in the model have to be determined by non-linearly fitting the experimental data to model, which may cause non-uniqueness of the parameters [12], [14]. For the equilibrium-interface model, it was developed by Little and Hodgson [13] and then improved by Deng et al. [14] by considering the convective effect through the air phase boundary layer. One of the main merits of this model lies in that, the characteristic (key) parameters used by this model, that is, the diffusion coefficient (*D*_m_), and the material/air partition coefficient (*K*), are the physical properties of the material-VOC pairs, which can be obtained by independent experiment. As far as the measurement of *D*_m_ and *K* is concerned, many kinds of methods have been proposed by virtue of sorption or emission test in environmental chambers, which can measure the *D*_m_ and *K* respectively or simultaneously. Tiffonnet et al. put forward a multi-sorption method to determine *K* [18]. Cox et al. proposed a microbalance method to obtain *K* and *D*_m_ [19]. For this method, *K* is pre-determined by sorption experiment in ventilated chamber and *D*_m_ is subsequently determined by fitting the experimental data to a mathematical model. Xu et al. applied a twin-chamber method to measure the *D*_m_ and *K*, respectively [20]. Li and Niu [21] proposed an inverse method to simultaneously determine the *D*_m_ and *K* by non-linearly fitting the experimental data to Deng and Kim’s model [22] with known initial emittable concentration and convective mass transfer coefficient. Xiong et al. developed a VVL method to determine the *K* by linear curve fitting [23]. However, there is still a challenge to determine the *D*_m_ and *K* based on the sorption process in airtight chamber.

Except for the characteristic parameters *D*_m_ and *K*, the convective mass transfer coefficient (*h*_m_) in the equilibrium-interface model introduced by Deng et al. [14] also affects the sorption behavior, especially for the initial period [24]. For the determination of *h*_m_, the empirical correlations based on heat and mass transfer analogy are widely used [14], [25], [26]. In addition, for some special cases, *h*_m_ can also be determined by directly studying the mass transfer process. For example, Zhang and Niu [27], [28] performed CFD simulation and experiment to analyze the mass transfer process in a field and laboratory emission cell (FLEC) and obtained a series of empirical correlations. However, these correlations are limited to FLEC, which are not applicable for other environmental chambers. And it should be pointed out that the uncertainty of applying correlations for calculation can reach up to ±20% or even ±25% for some cases [29]–[31]. Therefore, further improvement or accurate prediction of *h*_m_ aiming to specific cases is still needed.

The objectives of this study are to: (a) develop a novel method for simultaneously, rapidly and accurately measuring the diffusion coefficient, partition coefficient, as well as the convective mass transfer coefficient of formaldehyde and VOC in building materials by virtue of sorption process in airtight chamber; and (b) determine the convective mass transfer coefficient of semi-volatile organic compounds based on that of VOC.

## Methods

**Figure 1:**
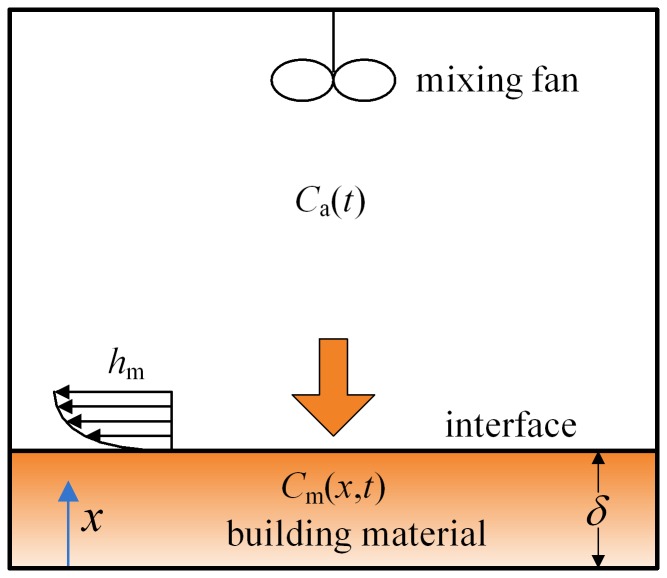
Schematic of formaldehyde and VOC sorption of building material in an airtight chamber.

A schematic of a building material placed in an airtight chamber (a chamber with an air exchange rate that is close to zero) with single surface sorption is shown in Figure 1. For the double surface sorption scenario, considering that the sorption characteristics in both surfaces are identical due to symmetry, we can treat the sorption process as single surface sorption in half of the building material. We assume that the building material is uniform, the formaldehyde and VOC diffusion process inside the building material is one dimensional and that the formaldehyde and VOC in the chamber are well mixed. These assumptions have been widely adopted in previous studies [32]–[35] and are further validated in the later context by the comparsion between the model prediction and experimental data.

To describe the principle mathematically, we denote the volume of the specimen as *V*_m_ and the volume of chamber as *V*_c_. The sample is put into the chamber which is then sealed. After that, a certain amount of formaldehyde and VOC is injected into the chamber, which generates a uniformly distributed chamber concentration *C*_a,0_ at sorption time *t* = 0. Then the sorption process occurs until equilibrium is reached.

If the equilibrium chamber formaldehyde and VOC concentration is *C*_equ_, the formaldehyde and VOC concentration in the material, *C*_m_, can be determined from Henry’s law:
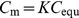
(1)where, *K* is the material/air partition coefficient.

Mass conservation of formaldehyde and VOC in the chamber gives:
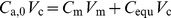
(2)


Then, *K* can be obtained by combining with equations (1) and (2):
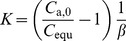
(3)where, *β* is defined as the ratio of material volume to chamber volume, *V*_m_/*V*_c_.

For the sorption process in an airtight chamber, based upon the afore-mentioned assumptions, the analytical solution can be derived as [15]:
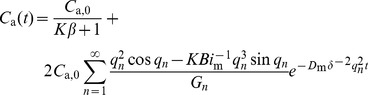
(4)where, *C*_a_ is the chamber formaldehyde or VOC concentration, µg/m^3^; *D*_m_ is the diffusion coefficient, m^2^/s; *Bi*_m_ is the Biot number for mass transfer ( = *h*_m_ *δ*/*D*_m_); *δ* is the thickness of the building material, m; *h*_m_ is the convective mass transfer coefficient, m/s; 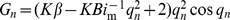
+ 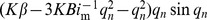, *n* = 1,2,…; *A* is the surface area of the building material, m^2^; *q_n_* are the positive roots of
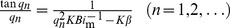
(5)Combining with equations (3) and (4), it yields:



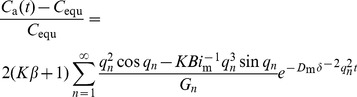
(6)For the infinite exponential series of equation (6), the terms decay very sharp. Therefore, for a sufficiently long sorption time, or the Fourier number (*Fo*_m_, defined as *D*_m_ *t*/*δ* ^2^ corresponding to the building material in an airtight chamber, and measure the real-time chamber formaldehyde and VOC concentration until equilibrium is reached;

1. Calculate *K* according to equation (3);
2. Based on the measured *C*_a_(*t*) and *C*_equ_, perform linear curve fitting and calculate SL and INT according to equation (10);
3. Set the existence interval of root *q*_1_ as [a, b] by using the bisection method;
4. Specify *q*_1_ = (a+b)/2;
5. Calculate *Bi*_m_ according to equation (5);
6. Define *f*(*q*_1_) as 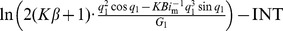 based on equation (9): if *f*(*q*_1_) is greater than 10^−12^, then go to step (4) and repeat; otherwise output *Bi*_m_ and *q*_1_;
7. Combine with equation (8) and the definition of *Bi*_m_ ( = *h*_m_ *δ*/*D*_m_), obtain the *D*_m_ and *h*_m_.

the sorption time) is equal to or higher than 0.125, only the first term (*n* = 1) is significant [36]. This means:
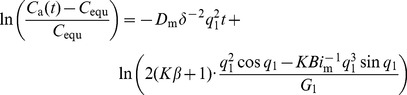
(7)where, *q*_1_ is the first root of equation (5); *G*_1_ is the first term of *G_n_*.

We denote the slope and intercept of equation (7) as SL and INT, respectively, that is:
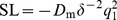
(8)

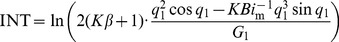
(9)


Then, equation (7) can be rewritten as:
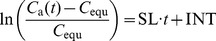
(10)


In this equation, the SL and INT are the functions of *K* (known, pre-determined by equation (3)), *D*_m_ (unknown) and *h*_m_ (unknown). Therefore, if the chamber formaldehyde and VOC concentrations are treated according to equation (10), the SL and INT can be obtained by virtue of linear curve fitting. Then, the two parameters *D*_m_ and *h*_m_ can be determined directly because we have two equations for SL and INT with two unknown parameters (*D*_m_ and *h*_m_). For the previous method [15], the SL and INT are used to determine *D*_m_ and *K* (*h*_m_ is pre-determined by correlations), while for this method, it is used to determine *D*_m_ and *h*_m_ (*K* is pre-determined by mass conservation), so they are quite different. Moreover, it should be pointed out that the traditional methods for prediction of *h*_m_ tend to be based on the empirical correlations originated from the heat and mass transfer analogy which is strongly related to sample geometry and the air flow across it [14], [25], [26]-it tends to be quite different from the application condition of the empirical correlation. For the present method, we overcome the problem and determine *h*_m_ based on the sorption process itself, i.e., the formaldehyde and VOC concentration data in the environmental chamber. Thus it is more accurate and can be regarded as a significant improvement of *h*_m_ measurement and of measurement precision for *K* and *D*_m_ of building materials. This method improves the application of the C-history for a closed (airtight) chamber [36] and is therefore called as the improved C-history method.

The detailed solving procedure for the determination of *K*, *D*_m_ and *h*_m_ is as follows:

1. Perform the sorption experiment for

### Analysis for Available Experimental Data in the Literature

Singer et al. [10], Huang et al. [37] performed a series of sorption experiments in airtight chambers. Their experimental data are used for applying the proposed method to determine the parameters *K*, *D*_m_ and *h*_m_. In Singer et al.’s experiment, the chamber contained many kinds of sorption building materials, such as walls and ceilings finished with gypsum wallboard, wood-veneer furniture, and so on. To simplify this complicated sorption problem, they consolidated all room surfaces into a single, conceptual material, and then used a unified adsorption/desorption rate (sorption) model to deal with the sorption into different material surfaces and obtained some equivalent parameters. The same idea is also applied here. The building material in the chamber is approximately treated as with area of 122.2 m^2^ (the sum of all material surface area) and thickness of 0.0254 m (0.0095 m+0.0159 m, the thickness of wallboard because it occupies most of the area), and is marked as wallboard in Table 1. Two kinds of pollutants in the experiment, i.e., toluene and TMB (1,2,4-TMBenzene) are taken for analysis. In Huang et al.’s experiment, just the experimental data of VOC mixture (methanol, isopropyl alcohol, ethyl acetate, toluene and cyclohexane, marked as TVOC for convenience in Table 1) in ceiling tile is available. The dimensions of the test ceiling tile is 27.9 cm×35.6 cm×1.3 cm (11inch×14inch×0.5inch) [38]. Both surfaces of the ceiling tile are exposed to air, and this scenario is treated as single surface sorption in half of the building material due to symmetry (with two surface area), as afore-mentioned. The details about the two experiments are summarized in Table 1.

**Table 1:** Parameters of the building material and chamber.

Material	Dimensions	Chamber volume	1/*β*	Description
Wallboard [10]	122.2 m^2^×2.54 cm	50 m^3^	16.1	Single surface sorption
Ceiling tile [33], [34]	27.9 cm×35.6 cm×1.3 cm	50 L	38.8	Double surface sorption

## Results and Discussion

### Determination of the Characteristic Parameters

**Figure 2:**
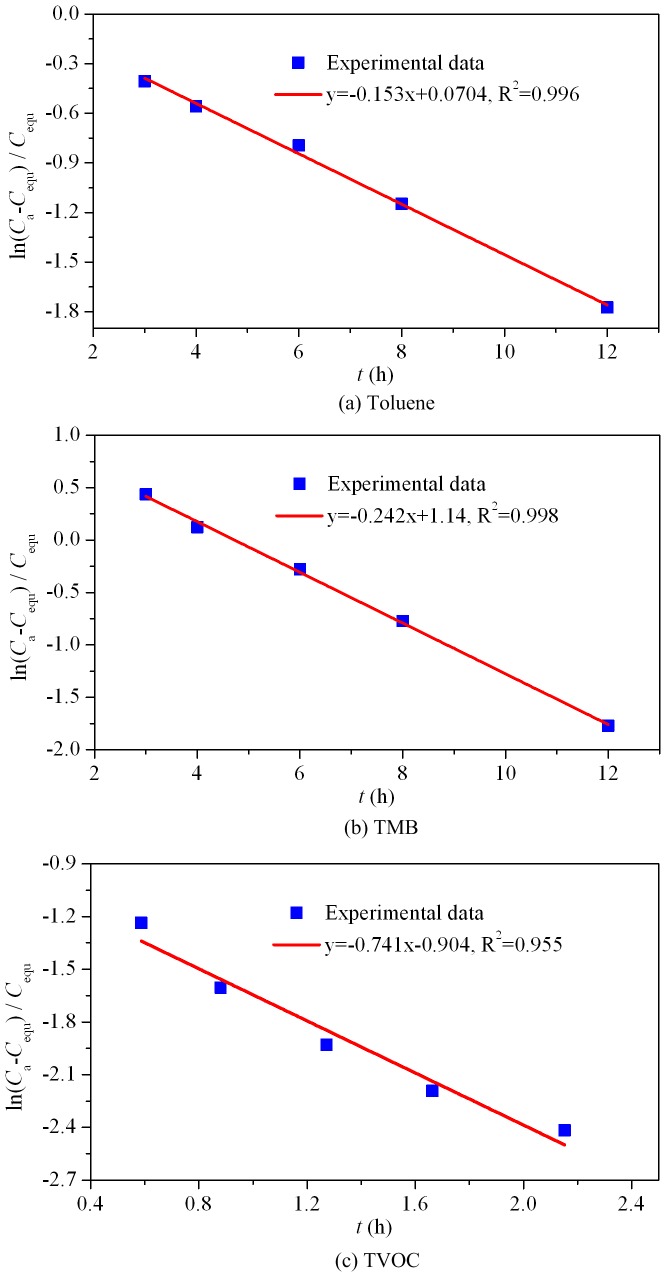
Linear curve fitting applying equation (10) for pollutants sorption in airtight chamber. (a) Toluene; (b) TMB; (c) TVOC.

In fact, Singer et al.’s [10] experiment contained three periods, i.e., sorption in airtight chamber, subsequently with flush process in ventilated chamber (air exchange rate: 4.8 h^−1^; duration: 1 h), and followed by desorption (re-emission) in airtight chamber, and just the first period is used for deriving the parameters *K*, *D*_m_ and *h*_m_. Experimental results from Singer et al. [10] and Huang et al. [37] indicate that the experimental time for the sorption process of test materials in airtight chamber to reach equilibrium is generally less than 30 hours, which is relatively rapid compared to the sorption process in ventilated chamber. Applying equation (10) to treat the experimental data and then performing linear curve fitting, the obtained results are shown in Figure 2.

Based on the afore-mentioned solving procedure, the characteristic parameters (*K*, *D*_m_) and *h*_m_ can be determined and the results are listed in Table 2, together with the square of the correlation coefficient (R^2^). According to ASTM Standard D5157-97 [39], a correlation coefficient (R) of 0.9 or greater can be regarded as generally indicative of adequate model performance. For the cases studied, all the R^2^ are greater than 0.95 (corresponding to R>0.97), which implies a high regression accuracy. By virtue of the measured *D*_m_ an *K*, the effective diffusion coefficient *D*_e_ ( = *D*_m_×*K*) can be calculated, and the results are also summarized in Table 2. This table reveals that the obtained *D*_e_ is in the order of 10^−7^ ∼ 10^−8^ m^2^/s, which is within the same order of some of the previous measurement results [40]–[42].

**Table 2:** Determined characteristic parameters for VOC sorption process.

Material	Pollutant	*D*_m_ (m^2^/s)	*K*	*D*_e_ (m^2^/s)	*h*_m_ (m/s)	R^2^
Wallboard	Toluene	6.78×10^−9^	2.42×10^1^	1.64×10^−7^	4.70×10^−5^	0.996
Wallboard	TMB	6.45×10^−9^	1.10×10^2^	7.10×10^−7^	7.27×10^−5^	0.998
Ceiling tile	TVOC	3.00×10^−9^	1.95×10^1^	5.85×10^−8^	9.45×10^−5^	0.955

### Validation of the Measured Parameters

**Figure 3:**
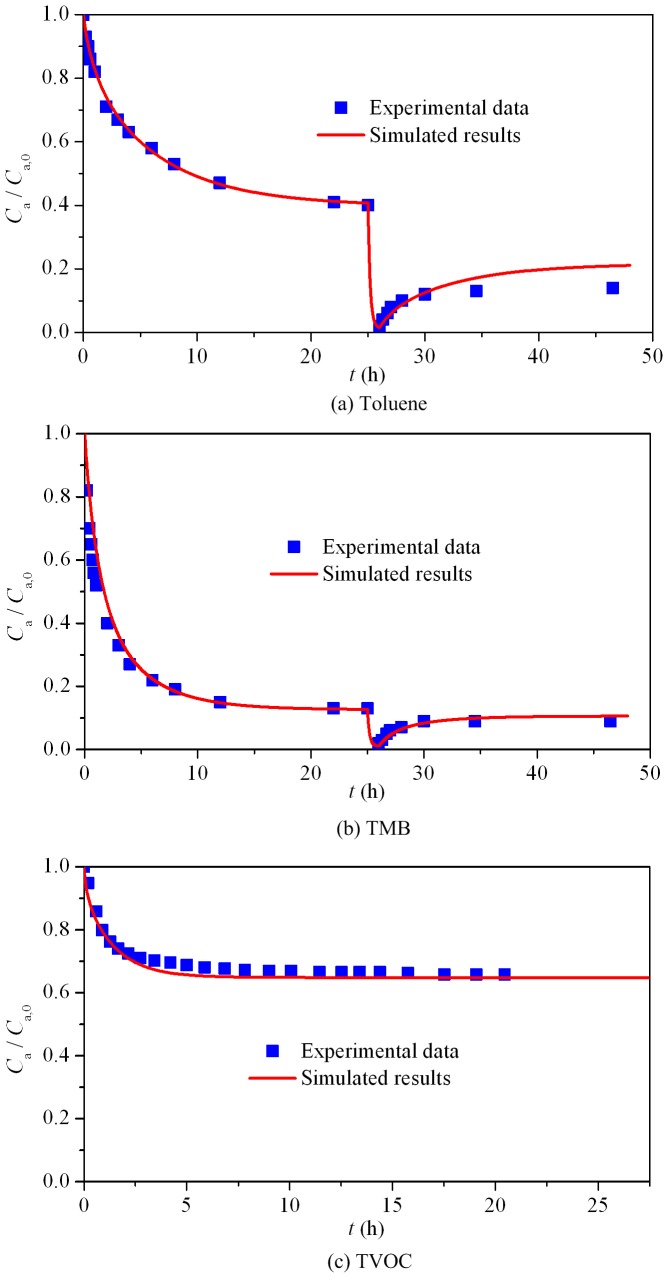
Comparison of simulated results with experimental data for different processes of pollutants in environmental chambers. (a) Toluene; (b) TMB; (c) TVOC.

If the sorption characteristic parameters (*K*, *D*_m_) and *h*_m_, are given, the sorption process of the material in airtight chamber can be simulated by applying a mass transfer model. For the proposed method, the parameters are not originated from direct nonlinear regression. Therefore, we can substitute the measured *K*, *D*_m_ and *h*_m_ from the present method into a mass transfer model to calculate the chamber VOC concentration, and then compare the calculated (simulated) results with the experimental data. This can be regarded as a convincing validation of the present method. For Singer et al.’s experiment, it contained three processes, and all the experimental data are used for validation. For this special case (three processes), Xu and Zhang’s model [22] can be used to sequentially simulate the chamber VOC concentration thus is applied. Figure 3 shows the comparison of the chamber VOC concentration between the simulated results (all use Xu and Zhang’s model) and the experimental data. For the sorption process in airtight chamber, the simulated results agree well with the experimental data, as indicated by Figures 3(a), (b) and (c). For the flush process in ventilated chamber and subsequent desorption (re-emission) process in airtight chamber, Figures 3(a) and (b) also represent good agreent between the simulation and the experiment, with just some deviations for toluene in the latter period of the desorption process. Considering that the flush and desorption processes are independent physical processes different from the sorption process used for determining *K*, *D*_m_ and *h*_m_, the good accordance under this condition effectively demonstrates that the measured characteristic parameters are accurate and reliable.

For the present improved C-history method, it firstly applies linear regression technique followed by solving equations to obtain the *K*, *D*_m_ and *h*_m_. The solution can be regarded as unique. While for the inverse method proposed by Li and Niu [21], a multi-parameter non-linearly regression technique is applied to determine the *D*_m_ and *K*. Since the *D*_m_ and *K* are coupled and are non-linearly fitted at the same time, there is a risk of having multiple solutions [14], [42].

### Impact of Measurement Uncertainty of Initial Chamber Concentration on the Determined Parameters

For the studied sorption problem, there is no formaldehyde or VOC existed in the material at the beginning of the sorption experiment. When the target VOC is injected into the chamber, the fan inside the chamber will mix the air and VOC, which generates an initial chamber VOC concentration *C*_a,0_. Considering that the tested chambers in the literature [10], [37] were constructed and operated based on ASTM standard D6670-01 [43] and D5116-97 [44], the mixing level can be regarded as satisfactory (higher than 80%) and the non-uniformity of concentration is generally less than 5%. That is to say, the mixing fan in the chamber can maintain satisfactory initial uniform VOC concentration in real experiment. However, for the sorption experiment in an airtight chamber, there is a potential difficulty in measuring the initial chamber VOC concentration since the material will sorb VOC simultaneously when VOC is injected into the chamber, which will cause measurement uncertainty of *C*_a,0_. This measurement uncertainty due to sorption effect can be reduced when the time for sampling VOC is short. Assuming the measurement uncertainty of *C*_a,0_ is 5%, here we will analyze the impact of this experimental uncertainty on the estimated parameters, *K*, *D*_m_ and *h*_m_.

According to the error propagation theory, the standard deviation of the partition coefficient, SD*_K_*, can be directly calculated as follows based on equation (3):
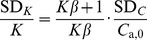
(11)where, SD*_C_* is the standard deviation of the initial chamber formaldehyde and VOC concentration, *C*_a,0_.

The standard deviation of the diffusion coefficient and the convective mass transfer coefficient can be obtained by numerical calculation. The procedure is: (1) add the standard deviation of the partition coefficient (SD*_K_*) to the partition coefficient; (2) use *K* ± SD*_K_* and run the same program to obtain the updated diffusion and convective mass transfer coefficients, which include the deviations; (3) compare the updated diffusion and convective mass transfer coefficients with the original determined parameters, and obtain the standard deviation.

Take TMB sorption in the wallboard as an example. With an experimental uncertainty (standard deviation) of 5% for the *C*_a,0_ as above-mentioned, the obtained standard deviations for the *K*, *D*_m_ and *h*_m_ are 6.36%, 2.79% and 4.81%, respectively. It indicates that the maximum standard deviation under this condition is less than 10%, which verifies the reliability of the present method.

### Potential Application of Measuring h_m_ of SVOC

For the sorption or emission of formaldehyde and VOC in building material, the physical process is controlled by the internal diffusion process as well as the external convective process, which is very different from that of the semi-volatile organic compounds (SVOC). For the latter, it is primarily subject to the external convective process (external control) [45], thus accurate prediction of *h*_m_ has a very important impact on the SVOC emission or sorption process. The traditional method for predicting *h*_m_ of SVOC is also based on the empirical correlations [45], which can be generally represented as:
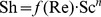
(12)where, Sh is the Sherwood number, *h*_m_ *δ*/*D*_a_; Sc is the Schmidt number, *ν*/*D*_a_; Re is the Reynolds number, *uδ*/*ν*; *ν* is the kinematic viscosity of the air, m^2^/s; *u* is the velocity of the air over the material, m/s; *D*_a_ is the diffusion coefficient of SVOC in the air, m^2^/s; *f*(Re) is the function of Re, which is related with the sample geometry and flow field; *n* is a parameter, it is usually taken as 1/3.

For different sample geometry and flow field inside the chamber, the expression of *f*(Re) should be different. And it is a challenge and long-term work to obtain the exact *f*(Re) under different conditions. Moreover, for some cases, the conditions might be quite different from the application conditions of the empirical correlations (i.e., *f*(Re) is different). These two factors lead to the primary error of correlations. Considering that, if we put samples with the same dimensions but emitting different pollutants (e.g., VOC and SVOC) in the same chamber, the expression of *f*(Re) should be the same due to the similar flow field inside the chamber. Then, the following formula can be derived based on equation (12):
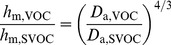
(13)where, *h*_m,VOC_ and *h*_m,SVOC_ are the convective mass transfer coefficients of VOC and SVOC, respectively; *D*_a,VOC_ and *D*_a,SVOC_ are the diffusion coefficients of VOC and SVOC in the air, respectively.

By virtue of equation (13), we can conveniently get *h*_m,SVOC_ from *h*_m,VOC_ rather than resorting to the exact expression of *f*(Re), which improves the measurement accuracy of *h*_m,SVOC_. It should be pointed out that *h*_m,VOC_ can be easily determined by performing similar VOC experiment applying the present improved C-history method. Compared to VOC, SVOC has much stronger sorption properties [45], [46]. SVOC is expected to sorb to particles and many surfaces including the surfaces of glass and stainless steel which are generally used to manufacture the experimental chamber. Previous research [45] indicated that the material/air partition coefficient of di-(2-ethylhexyl) phthalate (DEHP) from vinyl flooring owns the order of 10^11^, which is much higher than the partition coefficient of common VOC from building materials in indoor environment. Due to this significant partitioning of SVOC with different surfaces, it will take much longer time for SVOC to reach emission or sorption equilibrium both in chamber experiment and real environment. Experimental and numerical studies show that for SVOC emission from vinyl flooring, the experimental time is generally several months or even years [45], [47]. In addition, the common SVOC concentration in the chamber or indoor environment is very low, which leads to relatively long sampling time, taking about 24 h for one point at room temperature [47]. This analysis indicates that both the experimental time (several days) and sampling time (several minutes) of VOC sorption process are much less than that of SVOC. Therefore, the application of equation (13) derived by this paper can significantly reduce the time for *h*_m,SVOC_ measurement, thus will be very useful both for laboratory research and engineering application. Take a typical VOC, toluene, and a typical SVOC, DEHP sorption in wallboard as an example. The *D*_a_ of toluene and DEHP at 25°C are 7.74×10^−6^ m^2^/s [48] and 3.20×10^−6^ m^2^/s [49], respectively. If we use the improved C-history method to measure the *h*_m_ of toluene as 4.70×10^−5^ m/s, then we can predict the *h*_m_ of DEHP as 2.42×10^−5^ m/s based on equation (13).

**Figure 4:**
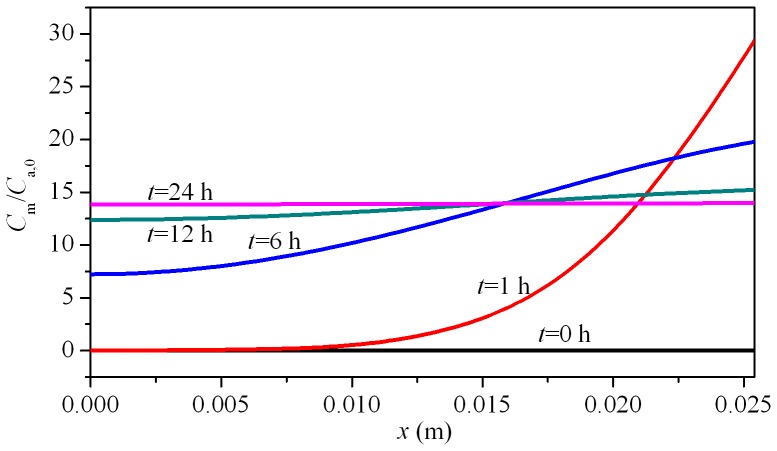
Distribution of TMB concentration in solid phase (*C*_m_) along with time.

### Analysis of Concentration Distribution Inside the Building Material

Theoretically, the present method heavily relies on the properties of the material being uniform with depth. And equation (2) also has an assumption that the formaldehyde and VOC concentration distribution is uniform with depth. Therefore, it is necessary to analyze the formaldehyde and VOC concentration distribution inside the material during the experiment. With the sorption of formaldehyde and VOC in the building material, a concentration gradient inside the solid or material phase does exist but decreases towards zero when approaching equilibrium. Take the TMB sorption in wallboard as an example for analyzing. Figure 4 shows the simulated TMB concentration distribution in the solid phase (*C*_m_) based on the determined characteristic parameters in Table 2. To quantify the *C*_m_ difference in depth, a parameter, *η*, is introduced to describe the uniformity of *C*_m_ [50]:
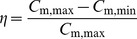
(14)where, *C*_m,max_, *C*_m,min_ are the maximum and minimum TMB concentrations in the solid phase, respectively. Obviously, *η* varies with the sorption time.

At the beginning (*t* = 0 h), *η* is equal to zero. With the sorption of TMB in the building mateiral, *η* first increases and then decreases, as shown in Figure 4. At the experimental time of 24 h, *η* is 0.97%, which is quite small. Therefore, when the sorption process approaches the equilibrium state, it is reasonable to appy uniform concentration distribution in the solid phase.

**Figure 5:**
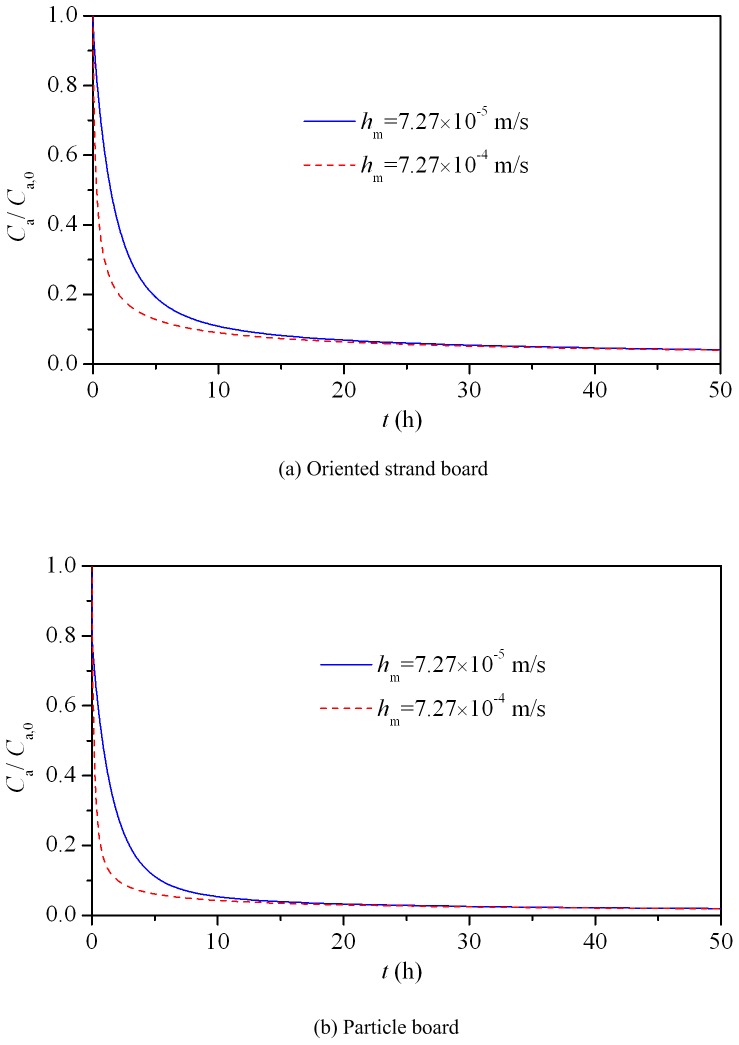
Sorption equilibrium analysis for hexanal in two kinds of materials. (a) Oriented strand board; (b) Particle board.

### Analysis of Applicability of the Proposed Method for Other Materials

The characteristic parameters (*D*_m_ and *K*) are generally different for different materials, which is partly due to the discrepancy in the microstructure. These two parameters, together with the convective mass transfer coefficient, material and chamber dimensions, play a significant impact on the experimental time of a material to reach sorption equilibrium. For other materials (not studied in this paper), if it takes too long time to reach the sorption equilibrium (e.g., longer than 7 days), the proposed method will not be a good choice for the determination of *K*, *D*_m_ and *h*_m_. Otherwise, it can be regarded as effective. Two other widely used materials, i.e., oriented strand board and particle board, are taken for analyzing the applicability of the improved C-history method. To simulate the sorption process of these two materials, the characteristic parameters should be given. Yuan et al. [51] measured the characteristic parameters of hexanal in oriented strand board (*D*_m_ = 4.20×10^−12^ m^2^/s, *K* = 6.60×10^3^), and Yang et al. [52] measured the characteristic parameters of hexanal in particle board (*D*_m_ = 7.65×10^−11^ m^2^/s, *K* = 3.29×10^3^). These parameters are taken for analysis. For other parameters in the simulation, they are selected as the same with that of TMB sorption in the wallboard. Figures 5 (a) and (b) show the simulated chamber hexanal concentration changing with the sorption time for oriented strand board and particle board, respectively (*h*_m_ = 7.27×10^−5^ m/s). It indicates that the time for these two materials to reach sorption equilibrium is about 2 days, which is relatively short. Therefore, the improved C-history method can be applicable for measuring the characteristic parameters of formaldehyde and VOC in oriented strand board and particle board. For the applicability of some other materials, further analysis is needed and the procedure is similar to that of oriented strand board and particle board. If we want to accelerate the sorption process to shorten the equilibrium time, one pathway is to increase the convective mass transfer coefficient, which can be realized by speeding up the mixing fan in the chamber. Figure 5 shows the simulated results when *h*_m_ changes from 7.27×10^−5^ m/s to 7.27×10^−4^ m/s. This figure reveals that the chamber hexanal concentration decreases sharply with large *h*_m_, and it takes less time to arrive at the equilibrium.

For the improved C-history method, it needs to sample a series of formaldehyde and VOC in the airtight chamber. Under this condition, if the chamber volume is large (e.g., 5∼50 m^3^), the multiple sampling volume can be regarded as negligible compared to the chamber volume, and the traditional instruments, such as GC/MS, HPLC, can still be applied. However, if the chamber volume is small (e.g., 10∼100 L), the multiple sampling volume will be large and thus will affect the mass conservation of formaldehyde and VOC in the chamber, and will further result in measurement errors. For the latter condition, just some special sampling or analyzing instruments can be applied, e.g., solid-phase micro extraction technique (SPME) for which the sampling volume is very small, INNOVA (a real-time gas VOC analyzer) which does not destroy the pollutants.

### Conclusions

Based on the sorption process in airtight chamber and the analytical solution, this paper proposes a novel method to simultaneously, rapidly and accurately measure the diffusion coefficient, the partition coefficient and the convective mass transfer coefficient. Applying this method, the three parameters of toluene and TMB sorption in wallboard, and TVOC sorption in ceiling tile are determined. The good agreement between the simulated results based on the measured parameters and the experimental data validates the effectiveness of the method. For the cases studied, the experimental time (sorption process) in the literature is generally less than 30 hours, demonstrating the time-saving merit. A case should be pointed out that this paper proposes a new idea for the determination of convective mass transfer coefficients of VOC and SVOC rather than the generally applied empirical correlations, which provides an alternative pathway for parameter measurement. Further research will focus on extending this method to measure the sorption behavior of other material-VOC pairs, as well as for SVOC.
