# Correction: A Novel Method for Measuring the Diffusion, Partition and Convective Mass Transfer Coefficients of Formaldehyde and VOC in Building Materials

**DOI:** 10.1371/annotation/cac8045c-5526-4cf6-81cc-32038949c12a

**Published:** 2013-05-07

**Authors:** Jianyin Xiong, Shaodan Huang, Yinping Zhang

Due to typesetting errors in the article, there was text placed incorrectly in the Methods section. A correct version of the text of the Methods section is available here: http://www.plosone.org/corrections/pone.0049342.001.cn.tif

